# Supplementary material for: GUIdock-VNC: using a graphical desktop sharing system to provide a browser-based interface for containerized software
Source: Gigascience. 2017 Feb 24;6(4):1–6. doi: 10.1093/gigascience/giw013 (PMC5530313; doi:10.1093/gigascience/giw013)
Supplement: GIGA-D-16-00089_Original_Submission.pdf [file giw013_GIGA-D-16-00089_Original_Submission.pdf]

Manuscript (latex file) Click here to download Manuscript bmc_article.tex

TECHNICAL NOTE

GUIdock-VNC: Using a graphical desktop sharing system to provide a browser-based interface for containerized software

Varun Mittal, Ling-Hong Hung, Jayant Keswani, Daniel Kristiyanto, Sung Bong Lee and Ka Yee Yeung*

*Correspondence: kayee@uw.edu
Institute of Technology, University of Washington, Tacoma, 1900 Commerce St, 98402 Tacoma, United States of America
Full list of author information is available at the end of the article

Abstract

Background: Software container technology such as Docker can be used to package and distribute bioinformatics workflows consisting of multiple software implementations and dependencies. However, Docker is a command line based tool and many bioinformatics pipelines consist of components that require a graphical user interface.

Findings: We present a container tool called GUIdock-VNC that uses a graphical desktop sharing system to provide a browser-based interface for containerized software. GUIdock-VNC uses the Virtual Network Computing protocol to render the graphics within most commonly used browsers. We also present a minimal image builder that can add our proposed graphical desktop sharing system to any Docker packages, with the end result that any Docker packages can be run using a graphical desktop within a browser. In addition, GUIdock-VNC uses the Oauth2 authentication protocols when deployed on the cloud.

Conclusions: As a proof-of-concept, we demonstrated the utility of GUIdock-noVNC in gene network inference. We benchmarked our container implementation on various operating systems and showed that our solution creates minimal overhead.

Keywords: Research Reproducibility; Docker; Containers; Software; Graphical user interface; bioinformatics

Findings

Background

Modern workflows in computational fields such as bioinformatics consist of multiple software implementations each with their own set of dependencies. Software container technology such as Docker ($http://www.docker.com$), package the dependencies along with the software and provide a method to reproduce these complex pipelines on multiple hardware and cloud platforms. For example, BioShadock [1],

BioDocker and and Bioboxes [2] are two frameworks aimed at reproducibly deploying bioinformatics workflows using Docker containers.

Many bioinformatics pipelines have a component which requires a Graphical User Interface (GUI) that can potentially limit the portability of the Dockerized workflows as different platforms use different methodologies to render the GUI. We have previously described and implemented GUIdock-X11 [3], an X11 based methodology for portably supporting GUI applications in containers on different platforms. While the X11 based display method can be conveniently deployed in the local environment by exposing a file socket from a container, deploying the image on a cloud and accessing it remotely is non-trivial. In addition, on systems such as Windows where there is no native X11 support, additional client software must be installed by the user to render the X11 graphics locally. Here we describe GUIdock-VNC which implements an improved browser-based solution which does not require the user to map ports, configure firewalls or install any additional specialized software.

GUIdock-VNC uses the Virtual Network Computing (VNC) protocol [4] to render the graphics. Instead of transferring commands and allowing a local client to render the graphics, VNC transfers a pre-rendered screen. Bandwidth requirements are minimized by only transferring the differences between the current screen and the last screen. This can actually be less chatty than the X11 methodology which is constantly sending display commands. noVNC is a browser-based VNC client implemented using HTML5 Canvas and WebSockets [5]. All modern browsers can use the HTML5 based noVNC client to display the screen locally. The browser transparently downloads the noVNC client from the container and becomes the terminal, thus eliminating the need for the user to configure and install separate software. This is a major advantage as the users of bioinformatics workflows are not necessarily technically trained in configuring computer systems.

Most importantly, GUIdock-VNC also facilitates the deployment of Docker applications on the cloud. With a browser based solution, we also have access to web-based authentication protocols such as Oauth2 [6] which allows for passwordless authentication using an email account. The host service is accessed and authenticated through the HTTP/HTTPS port, greatly simplifying the configuration necessary to support cloud based platforms.

Our contributions

We implemented GUIdock-VNC which adds and configures a software layer inside a Docker container to allow applications to export a GUI using the VNC protocol. When deployed on the cloud, authentication is provided using Oauth2. In addition, we provide a set of minimal base images to allow the users to add the host graphical desktop interface to any existing Dockerfiles. No client software installation is necessary for the users as GUIdock-VNC uses the HTML5 noVNC browser-based client to display the GUI. All our tools are publicly available on github.

We benchmarked the implementation on a real-world bioinformatics pipeline. Our results showed that noVNC creates minimal overhead and GUIdock-VNC is superior to our previous work GUIdock-X11 [3] and other virtual machine based deployment solutions.

Related work

Software containers and Docker

A software container packages an application with everything it needs to run, including supporting libraries and system resources. Containers differ from traditional virtual machines (VMs) in that the resources of the operating system (OS) and not the hardware are virtualized. In addition, multiple containers share a single OS kernel, thus saving considerable resources over multiple VMs.

Linux has supported OS level virtualization for several years. Docker (*https : //www.docker.com/*) is an open source project that provides tools to setup and

deploy Linux software containers. While Docker can run natively on Linux hosts, a small Linux VM is necessary to provide the virtualization services on Mac OS and Windows systems. On non-Linux systems, a single Docker container consists of a mini-VM, the Docker software layer and the software container. However, multiple Docker containers can share the same mini-VM, saving considerable resources over using multiple individual VMs. Recently, support for OS level virtualization has been added to Windows and the Macintosh operating systems (Mac OS). Beta versions of Docker for both Windows and Mac OS are now available that allow Docker to run natively. Subsequently, these beta versions allow native Windows and Mac OS software to be containerized and deployed in a similar manner [7]. Docker containers therefore provide a convenient and light method for deploying open-source workflows on multiple platforms.

GUIDock-X11

Although Docker provides a container with the original software environment, the host system, where the container software is executed, is responsible for rendering graphics. Our previous work, GUIdock-X11 [3], is one of the solutions in bridging the graphical information from user and Docker containers by using the X11 common graphic interface. GUIdock-X11 passes the container X11 commands to a host X11 client which renders the GUI. Security is handled by encrypting the commands through secure shell (ssh) tunneling. We demonstrated the use of GUIdock-X11 [3] for systems biology applications, including Bioconductor packages written in R, C++ and Fortran, and Cytoscape, a stand-alone java-based application with a graphical user interface. Neither Windows nor Mac OS use X11 natively to render their graphics. Additional software such as MobaXterm [8] or SoCat [9] are needed to emulate X11 and locally render the graphics commands exported by the Docker container. However, a major advantage of the X11 method is that the commands

to render the graphics and not the graphics themselves are transmitted, potentially reducing the total bandwidth required.

Table 1 summarizes the differences between GUIdock-VNC and our previous work GUIdock-X11.

Table 1 Comparison between GUIdock-X11 and GUIdock-VNC

**Feature**	**GUIdock-X11**	**GUIdock-VNC**
**Can be deployed on phones/tablets?**	No	Yes
**Security**	ssh-tunnel	OAuth2
**Bandwidth**	Low	Low to Medium
**Cloud integration difficulty**	Medium	Simple
**Dockerfile setup**	Manual editing	Automatic conversion of base Docker images

Case study: Inference of gene networks

The inference of gene networks is a fundamental challenge in systems biology. We use gene network inference as a case study to demonstrate that GUIdock-X11 and GUIdock-VNC can be used to yield reproducible results from bioinformatics workflows. We have previously developed inference methods using a regression-based framework, in which we search for candidate regulators (i.e. parent nodes) for each target gene [10, 11, 12]. Our methods are implemented in R, C++, and Fortran, and the implementation is available as a Bioconductor package called *networkBMA* [13] (*http://bioconductor.org/packages/release/bioc/html/networkBMA.html*). In order to visualize the resulting gene networks, we previously developed a Cytoscape app called CyNetworkBMA [14], (*http://apps.cytoscape.org/apps/cynetworkbma*). Cytoscape is a Java-based stand-alone application with a GUI to analyze and visualize graphs and networks [15, 16, 17]. Our app, CyNetworkBMA [14], integrates our *networkBMA* Bioconductor package into Cytoscape, allowing the user to directly visualize the resulting gene networks inferred from *networkBMA* using the Cytoscape utilities. The integration of multiple pieces of sofware, each with its own

software dependencies, make CyNetworkBMA an ideal proof-of-concept application for the illustration of the utility of GUIdock-VNC.

Implementation of GUIdock-VNC

Figure 1 shows an overview of GUIdock-VNC.

Figures/Architecture.pdf

Figure 1 Architecture overview of GUIdock-VNC. In the proposed architecture, each container is a self-contained web-server that can be accessed using a single port. When deployed on the cloud, the services can be accessed using the cloud provider's network address translation (NAT) mechanism. Each container is also capable of OAuth2 authentication that can be enabled while deploying the application. Once enabled, the user will be required to sign-in through an identity provider (such as Google in the current prototype). After the authentication, the user browser will be automatically redirected to the application.

Virtual Network Computing (VNC)

VNC is a framebuffer based protocol that was written to view and control remote desktop over the internet [4]. VNC is essentially a server program that attaches to a display server like X11 and creates a proxy between the client and the display server. The proxy server takes in input from the client and relays it to the display server while at the same time the display server sends pre-rendered display images to the client. VNC is thus a network intensive protocol, although the amount of data transferred back and forth can be reduced by using various compression technologies on the transfer layer. In our implementation, we use Xvnc/Xvfb (X virtual frame buffer) [18] to provide a lightweight VNC/X11 display server.

noVNC

noVNC is a browser based VNC client [5]. The name *noVNC* means that the traditional VNC client is not needed, and that a modern browser with HTML5 and websockets support can be used to access and control a remote VNC server. This noVNC technology is particularly interesting as almost all browsers, both desktop

and mobile versions have HTML5 extensions built-in. An additional layer on the host is required for the VNC server to communicate through websockets. We use nginx ($https://www.nginx.com/$), a fast and light reverse-proxy web server for this purpose.

Authentication methods: Oauth2

For containers that are not deployed locally, i.e. on a network or cloud, security is a concern as the traffic between the viewer and server can be seen by anyone with access to the network. Oauth2 [6] is an authentication method that is commonly used by major corporations to validate third party applications. Specifically, Oauth allows users to log onto third party websites using their existing Google, Twitter or Facebook accounts, thus avoiding the creation of additional accounts for the third party websites. In the present era, where it is extremely difficult to host identity services and securing communication, public authentication services like Oauth2 plays an important role. Providers like Google, Facebook, LinkedIn can be used to validate any user registered with an email at one of these websites. In our prototype we have created a prototype for Google identity server. Each container can be forced to login through one of these public providers.

Automatic conversion of base Docker images

To add the noVNC graphical desktop to a Docker image, the converted image requires a web-server, a headless display server running inside the container and a VNC server (see Figure 2). The webserver is required to serve javascript based noVNC client and the headless display server routes all drawing instructions to the VNC server which are then sent to the client running the browser based noVNC javascript client. Due to the three active components running inside the container, we generate a bash script to work as the entry point for the container. Therefore in order to assist users and to let them start application dependent services we have created a tool to bootstrap standard images with noVNC capability. The

tool accepts a json script as input with defined parameters for files to be copied, additional software to be installed (using apt-get) and the location of the startup script which is then tied into the entrypoint.

The tool is extensible and can be used to define all dockerfile parameters as standard run commands.

Figures/Infrastructure.pdf

Figure 2 Services running inside container. Apart from user applications, there are two web services running inside the container and a reverse proxy (Nginx) to act as an interface for the container. The first web-service is the noVNC interface connected to the VNC server. The noVNC server is a python plus javascript application framework used for establishing web-sockets for VNC packet interchange. The second web-service is an optional broker service that helps in exchanging data through a datastore interface (Mongodb) and a message passing queue (RabbitMQ).

Applications

We illustrated the utility of GUIdock-VNC in a proof-of-concept case study of gene network inference. Specifically, we applied GUIdock-VNC to a RNAseq dataset consisting of 675 human cancer cell lines [19]. We downloaded the variance stabilized version of the normalized RNAseq data, and extracted a subset of 84 genes that belong to 21 cancer-related pathways (see Supplementary Table 12 in Klijn *et al.* [19]). We applied the ScanBMA [12] gene network inference algorithm as implemented in the CyNetworkBMA app from within the GUIdock-VNC container.

We show that we get identical results after deploying the package across different browsers on different operating systems. Figure 3 shows screenshots of using (a) Internet Explorer on Windows 8.1, (b) Google Chrome on Ubuntu Linux, (c) Safari on MacOS, and (d) Google Chrome on Android. To summarize, we demonstrate reproducibility of analytical results when GUIdock-VNC is deployed on different browsers and different operating systems.

Figures/Screenshots.pdf

Figure 3 CyNetworkBMA on various browsers and operating systems: (a) Internet Explorer on Windows 8.1, (b) Google Chrome on Ubuntu Linux, (c) Safari on MacOS, and (d) Google Chrome on Android. Given that the interaction to the container is made through a browser, any device with a browser supporting HTML5 with consistent user experience and results, even on mobile devices such as Android and iOS devices. In the case of mobile devices, the Docker container runs on remote machines (e.g. on a VM instance on cloud).

Benchmarking computational efficiency

Since we have added extra services to the container, it is essential to investigate the performance overhead introduced by these additional services and the container. We conducted an extensive empirical study on comparing performance over different platforms with different hypervisors using GUIdock-X11 and GUI-VNC. As a baseline, we compared the performance of running GUIdock-X11 and GUIdock-VNC on Docker containers to running the CyNetworkBMA app natively. In addition, we also compared our results to running the CyNetworkBMA via a virtual machine (VM). We experimented the performance of each of these four options (native, GUIdock-X11, GUIdock-VNC, VM) on the Linux, Macintosh and Windows operating systems.

In our benchmarking experiments, we used the time series data of the first network from the DREAM 4 crowd sourcing challenge [20, 21]. This simulated dataset consists of 100 genes across 21 time points. In order to account for variability in our empirical experiments, we repeated each configuration, *i.e.* each (OS, option) pair, four times. These replicated experiments are represented by ”RUN1”, ”RUN2”, ”RUN3”, ”RUN4” in Table 2. In addition, we added warm-up runs to ensure steady-state execution time.

Table 2 shows a consistent minimal overhead of running the proposed container, which is only marginally higher than running application natively. In particular, we computed the ratio of the average execution time over the 4 runs to the ”native”

baseline execution time. Figure 4 shows the ratio of the average execution time of each of GUIdock-X11, GUIdock-VNC and VM to the baseline "native" on the Linux, MacOS and Windows operating systems. On the Linux, MacOS and Windows operating systems, we observed comparable execution time for both GUIdock-X11 and GUIdock-VNC.

Table 2 Execution time in empirical study across the Linux, Macintosh and Windows operating systems. "Native" means running the CyNetworkBMA app natively on the corresponding OS. "VM" means running the CyNetworkBMA app from a virtual machine on the corresponding OS. The column "average" is the average execution time over the 4 runs. The column "ratio" is the ratio of the average running time to the "native" baseline.

Platform	Environment	RUN 1	RUN 2	RUN 3	RUN 4	Average	Ratio
Linux	Native	104	100	97	102	100	1
	GUIdock-X11	130	131	131	130	130	1.29
	GUIdock-VNC	134	133	133	134	134	1.39
	VM	187	185	186	185	186	1.92
MacOS	Native	97	97	96	96	96	1
	GUIdock-X11	120	116	118	123	119	1.23
	GUIdock-VNC	123	121	120	124	122	1.26
	VM	148	143	151	150	148	1.54
Windows	Native	125	127	130	128	127	1
	GUIdock-X11	155	157	155	156	155	1.22
	GUIdock-VNC	157	160	162	161	160	1.25
	VM	179	179	182	184	181	1.42

Figures/execRatios2.png

Figure 4 The bar graph shows the ratio of the average execution time to the baseline of running CyNetworkBMA natively for each of Linux, MAC OS and Windows. The first value for each platform is the ratio of the native execution runtime to itself, and therefore is always equal to 1. The remaining three values correspond to the ratio of the average execution time for GUIdock-X11, GUIdock-VNC and VM respectively to the baseline "native".

Discussion

We present a container tool called GUIdock-VNC that uses a graphical desktop sharing system to provide a browser-based interface for containerized software. The merits of GUdock-VNC are summarized as follows:

No installation on client side. Our proposed container GUIdock-VNC is a self contained display server with user application and a HTML5 based VNC client,

which can be accessed using the web-browser. Therefore, any HTML5 capable browser is sufficient on the client side. Almost all modern popular browsers such as Mozilla Firefox, Webkit (Apple Safari and Google Chrome), Microsoft Edge support HTML5 extentions.

Mobile capable. Since HTML5 extentions that are required to access noVNC are also supported on mobile versions on modern web-browsers, our GUIdock-VNC solution is also accessible through a mobile device such as phones and tablets. The container is a stateful solution, such that it preserves session information even after a disconnected user session. Therefore, researchers can access the container on the go and subsequently continue working on the workstation in the labs.

Cloud integration. There is a self-contained webserver inside the GUIdock-VNC container which is required to access the applications. Our GUIdock-VNC container can be hosted on the cloud by using a reverse proxy or simple NAT (Network Address Translation) rule to passon any incoming request to the container. We tested the solution with major cloud vendors such as Amazon AWS, Google Cloud and Microsoft Azure and the NAT forwarding works without any network interference.

Security using oAuth2. To ensure secure access to the container, we are using OAuth2 from Google (replacable by any other identy provider such as LinkedIn, Facebook, Twitter etc.) to authenticate users by ensuring the identity registered with container is owned by the user requesting container access. This can be done by providing email id, provider user id and secret password as parameters to the container. If these parameters are provided while initiating the container, the broker will redirect the user to the identity provider to verify the email address. Once authenticated the identity provider redirects the user to the container.

Availability and requirements

- Project name: GUIdock-VNC
- Project home page: $https://github.com/biodepot$

- Contents available for download: Docker Images, Dockerfiles, installation scripts and execution scripts.
- Operating system(s): Linux, Mac OS X, Microsoft Windows. Specifically, we tested GUIdock-VNC on
  - Linux: Fedora, Ubuntu 15.04
  - Mac OS X: 10.9, 10.10
  - Microsoft Windows: 7, 8.1, 10
  - Android, IOS
- Programming language(s): Python, HTML, JavaScript
- Browsers tested
  - Google Chrome
  - Firefox
  - Safari
- License: MIT License

Abbreviations

GUI: graphical user interface NAT: network address translation VM: virtual machine VNC: Virtual Network Computing

Declarations

Acknowledgments

Varun Mittal, Ling-Hong Hung and Ka Yee Yeung are supported by NIH grant U54-HL127624. We would like to thank Microsoft Azure for computing resources. Daniel Kristiyanto is sponsored by the U.S. Department of State and American-Indonesian Exchange Foundation (AMINEF) through Fulbright Scholarship, and gratefully acknowledges funding provided by the University of Washington in the form of full tuition waivers.

Competing interests

The authors declare that they have no competing interests.

Author's contributions

VM is the primary developer for GUIdock-VNC. KYY coordinated the manuscript preparation. VM, LHH and KYY drafted the manuscript. LHH designed the benchmarking experiments. VM, LHH, DK, JK and SBL performed the benchmarking experiments. DK and SBL contributed to the comparison of GUIdock-VNC and GUIdock-X11. VM, JK and SBL contributed to the writing of the user manual. VM and DK made the videos in additional data files. All authors tested GUIdock-VNC, read and approved the final manuscript.

References

1. Moreews, F., Sallou, O., Ménager, H., Le Bras, Y., Monjeaud, C., Blanchet, C., Collin, O.: Bioshadock: a community driven bioinformatics shared docker-based tools registry. F1000Research **4**, 1443 (2015)
2. Bio Docker: Docker for Bioinformatics. http://biodocker.org/
3. Hung, L.-H., Kristiyanto, D., Lee, S.B., Yeung, K.Y.: Guidock: Using docker containers with a common graphics user interface to address the reproducibility of research. PLOS One **11**(4), 0152686 (2016)
4. Richardson, T., Stafford-Fraser, Q., Wood, K.R., Hopper, A.: Virtual network computing. IEEE Internet Computing **2**(1), 33 (1998)
5. noVNC by kanaka. http://kanaka.github.io/noVNC/
6. OAuth. http://oauth.net/
7. Docker for Mac and Windows Beta: the simplest way to use Docker on your laptop. https://blog.docker.com/2016/03/docker-for-mac-windows-beta/
8. MobaXterm: Enhanced terminal for Windows with X11 server, tabbed SSH client, network tools and much more. http://mobaxterm.mobatek.net/
9. socat: Multipurpose relay (SOcket CAT). http://www.dest-unreach.org/socat/doc/socat.html
10. Yeung, K.Y., Dombek, K.M., Lo, K., Mittler, J.E., Zhu, J., Schadt, E.E., Bumgarner, R.E., Raftery, A.E.: Construction of regulatory networks using expression time-series data of a genotyped population. Proceedings of the National Academy of Sciences **108**(48), 19436–19441 (2011)
11. Lo, K., Raftery, A., Dombek, K., Zhu, J., Schadt, E., Bumgarner, R., Yeung, K.Y.: Integrating external biological knowledge in the construction of regulatory networks from time-series expression data. BMC Systems Biology **6**(1), 101 (2012)
12. Young, W.C., Raftery, A.E., Yeung, K.Y.: Fast Bayesian inference for gene regulatory networks using ScanBMA. BMC Systems Biology **8**(1), 47 (2014)
13. Yeung, K.Y., Fraley, C., Young, W.C., Bumgarner, R., Raftery, A.E.: Bayesian model averaging methods and R package for gene network construction. In: Big Data Analytic Technology For Bioinformatics and Health Informatics (KDDBHI), Workshop at the 20th ACM SIGKDD Conference on Knowledge Discovery and Data Mining (KDD) (2014)

14. Fronczuk, M., Raftery, A.E., Yeung, K.Y.: Cynetworkbma: a cytoscape app for inferring gene regulatory networks. Under revision

15. Shannon, P., Markiel, A., Ozier, O., Baliga, N.S., Wang, J.T., Ramage, D., Amin, N., Schwikowski, B., Ideker, T.: Cytoscape: a software environment for integrated models of biomolecular interaction networks. Genome Research **13**(11), 2498–2504 (2003)

16. Christmas, R., Avila-Campillo, I., Bolouri, H., Schwikowski, B., Anderson, M., Kelley, R., Landys, N., Workman, C., Ideker, T., Cerami, E., Sheridan, R., Bader, G.D., Sander, C.: Cytoscape: A Software Environment for Integrated Models of Biomolecular Interaction Networks, pp. 12–16 (2005). http://educationbook.aacrjournals.org/cgi/content/full/2005/1/12

17. Cline, M.S., Smoot, M., Cerami, E., Kuchinsky, A., Landys, N., Workman, C., Christmas, R., Avila-Campilo, I., Creech, M., Gross, B., Hanspers, K., Isserlin, R., Kelley, R., Killcoyne, S., Lotia, S., Maere, S., Morris, J., Ono, K., Pavlovic, V., Pico, A.R., Vailaya, A., Wang, P.-L., Adler, A., Conklin, B.R., Hood, L., Kuiper, M., Sander, C., Schmulevich, I., Schwikowski, B., Warner, G.J., Ideker, T., Bader, G.D.: Integration of biological networks and gene expression data using cytoscape. Nature Protocols, 2366–2382 (2007)

18. Xvfb: virtual framebuffer X server for X Version 11. https://www.x.org/releases/X11R7.6/doc/man/man1/Xvfb.1.xhtml

19. Klijn, C., Durinck, S., Stawiski, E.W., Haverty, P.M., Jiang, Z., Liu, H., Degenhardt, J., Mayba, O., Gnad, F., Liu, J., Pau, G., Reeder, J., Cao, Y., Mukhyala, K., Selvaraj, S.K., Yu, M., Zynda, G.J., Brauer, M.J., Wu, T.D., Gentleman, R.C., Manning, G., Yauch, R.L., Bourgon, R., Stokoe, D., Modrusan, Z., Neve, R.M., de Sauvage, F.J., Settleman, J., Seshagiri, S., Zhang, Z.: A comprehensive transcriptional portrait of human cancer cell lines. Nature Biotechnology **33**, 306–312 (2015)

20. Marbach, D., Schaffter, T., Mattiussi, C., Floreano, D.: Generating Realistic In Silico Gene Networks for Performance Assessment of Reverse Engineering Methods. Journal of Computational Biology **16**(2), 229–239 (2009)

21. Marbach, D., Prill, R.J., Schaffter, T., Mattiussi, C., Floreano, D., Stolovitzky, G.: Revealing strengths and weaknesses of methods for gene network inference. Proceedings of the National Academy of Sciences **107**(14), 6286–6291 (2010)

Additional Files

Additional file 1 — User manual for GUIdock-VNC.

Additional file 2 — Video of GUIdock-VNC demo

Available on Youtube $https://youtu.be/iaVPnLhOLg0$.

Additional file 3 — Video of deploying GUIdock-VNC on the cloud

Figure Captions

Figure 1

Architecture overview of GUIdock-VNC. In the proposed architecture, each container is a self-contained web-server that can be accessed using a single port. When deployed on the cloud, the services can be accessed using the cloud provider's network address translation (NAT) mechanism. Each container is also capable of OAuth2 authentication that can be enabled while deploying the application. Once enabled, the user will be required to sign-in through an identity provider (such as Google in the current prototype). After the authentication, the user browser will be automatically redirected to the application.

Figure 2

Services running inside container. Apart from user applications, there are two web services running inside the container and a reverse proxy (Nginx) to act as an interface for the container. The first web-service is the noVNC interface connected to the VNC server. The noVNC server is a python plus javascript application framework used for establishing web-sockets for VNC packet interchange. The second web-service is an optional broker service that helps in exchanging data through a datastore interface (Mongodb) and a message passing queue (RabbitMQ).

Figure 3

CyNetworkBMA on various browsers and operating systems: (a) Internet Explorer on Windows 8.1, (b) Google Chrome on Ubuntu Linux, (c) Safari on MacOS, and (d) Google Chrome on Android. Given that the interaction to the container is made through a browser, any device with a browser supporting HTML5 with consistent user experience and results, even on mobile devices such as Android and iOS devices. In the case of mobile devices, Docker run on other machines (e.g. on a VM instance on cloud).

Figure 4

The bar graph shows the ratio of the average execution time to the baseline of running CyNetworkBMA natively for each of Linux, MAC OS and Windows. The first value for each platform is the ratio of the native execution runtime to itself, and therefore is always equal to 1. The remaining three values correspond to the ratio of the average execution time for GUIdock-X11, GUIdock-VNC and VM respectively to the baseline ”native”.

Figure 1

Click here to download Figure Architecture.pdf

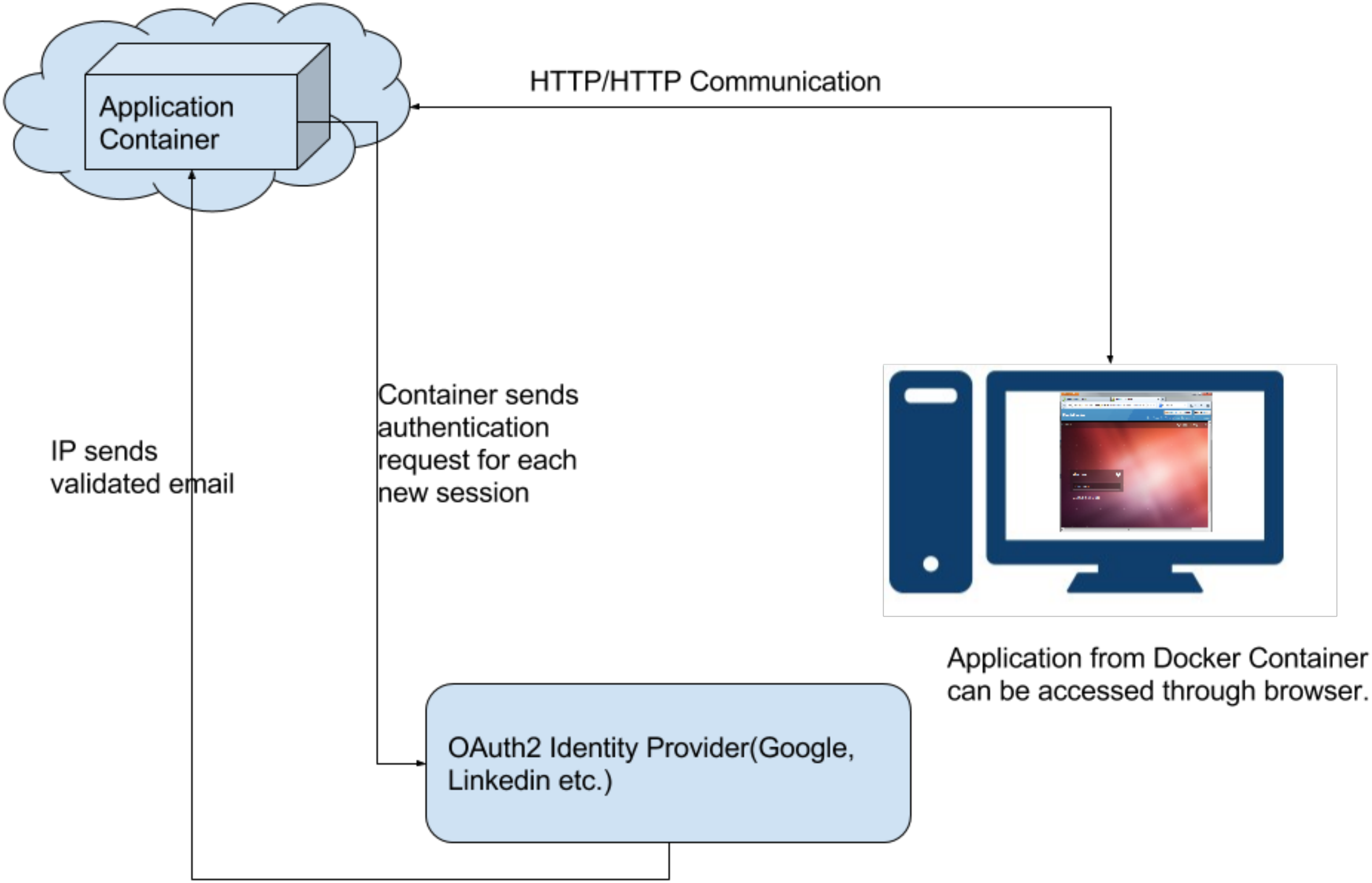

Figure 2

Click here to download Figure Infrastructure.pdf

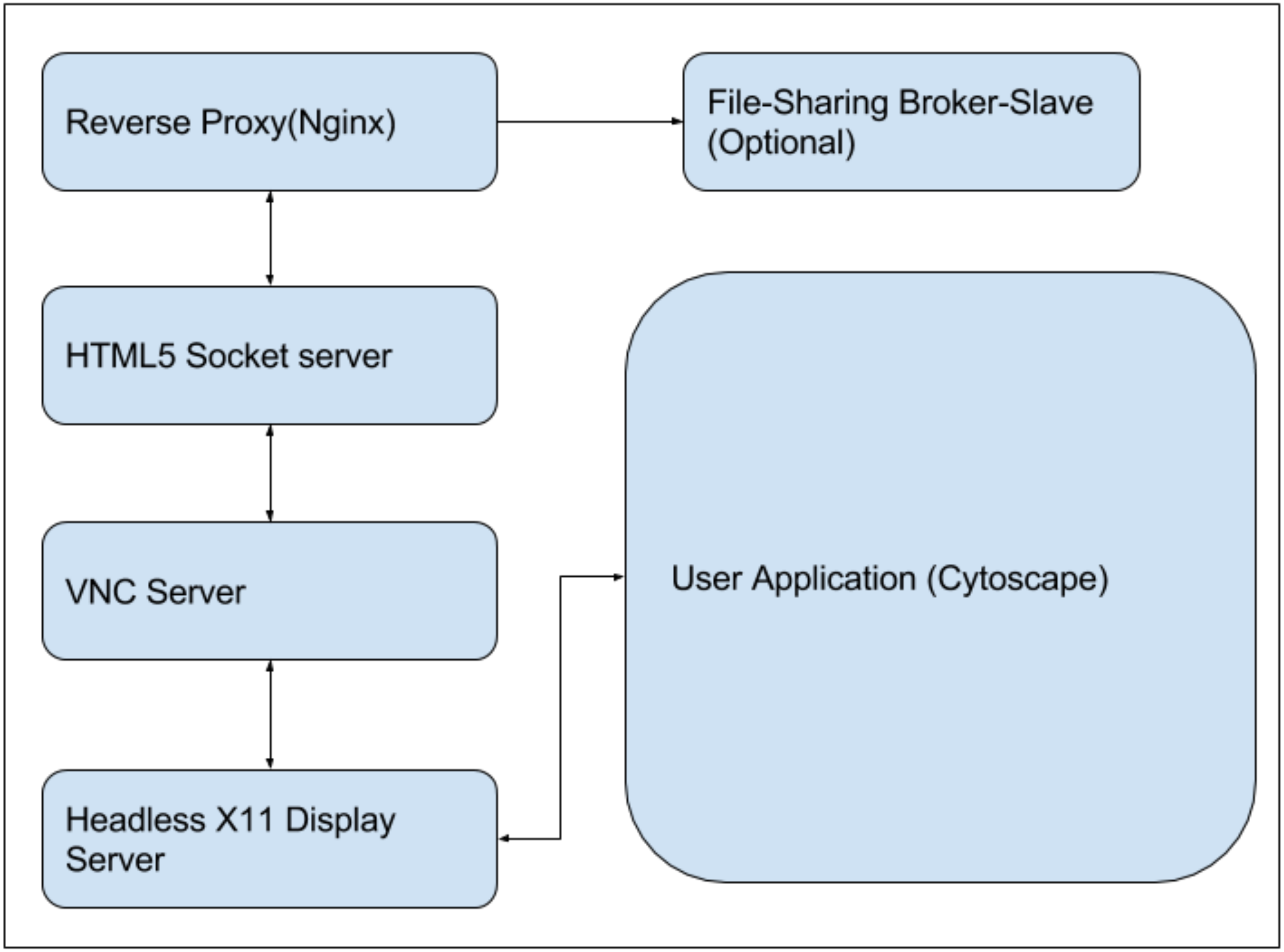

Docker Application Image

Figure 3

Click here to download Figure Screenshots.pdf

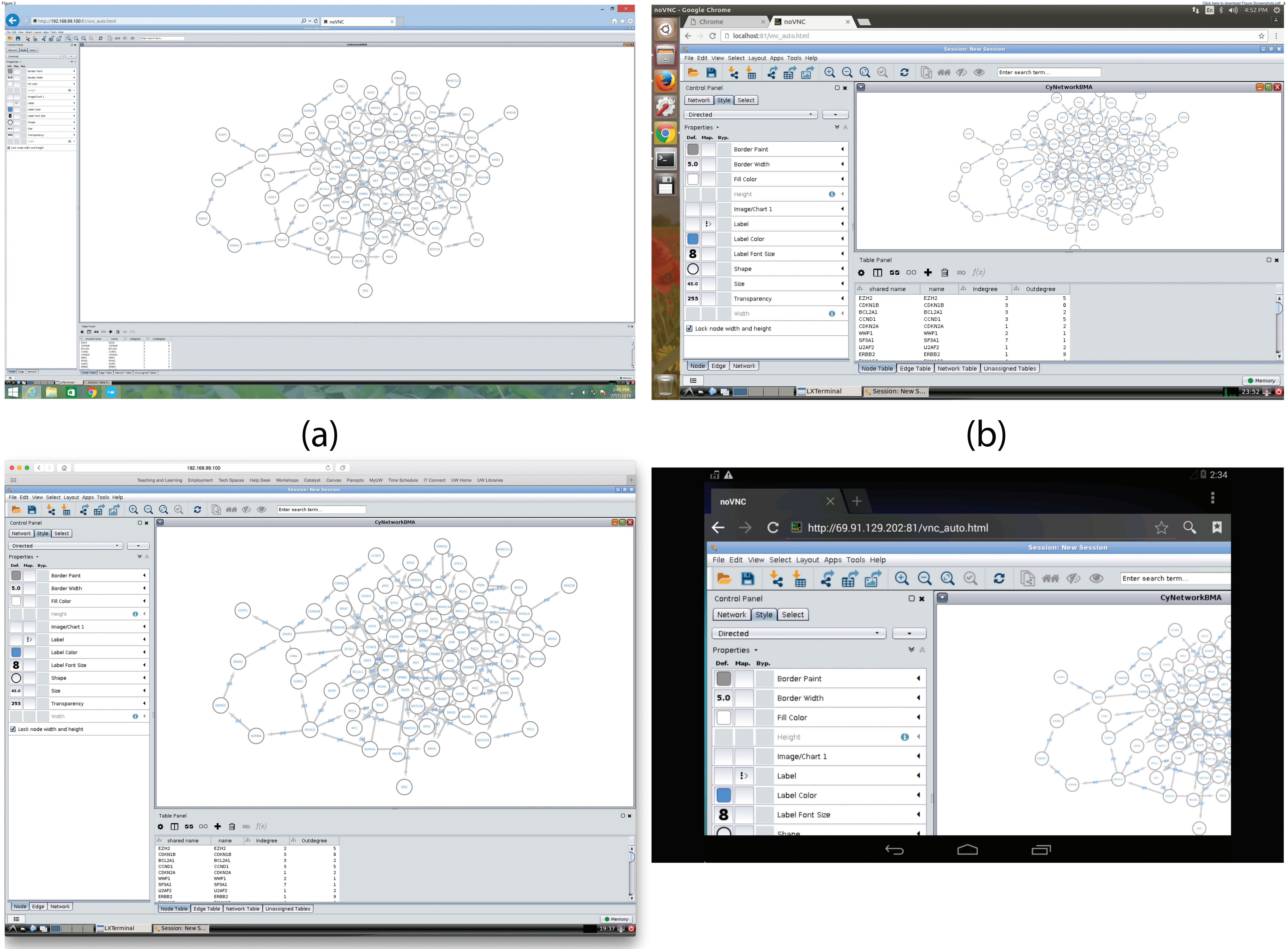

(a)

(b)

(c)

(d)

Figure 4

Click here to download Figure execRatios2.png

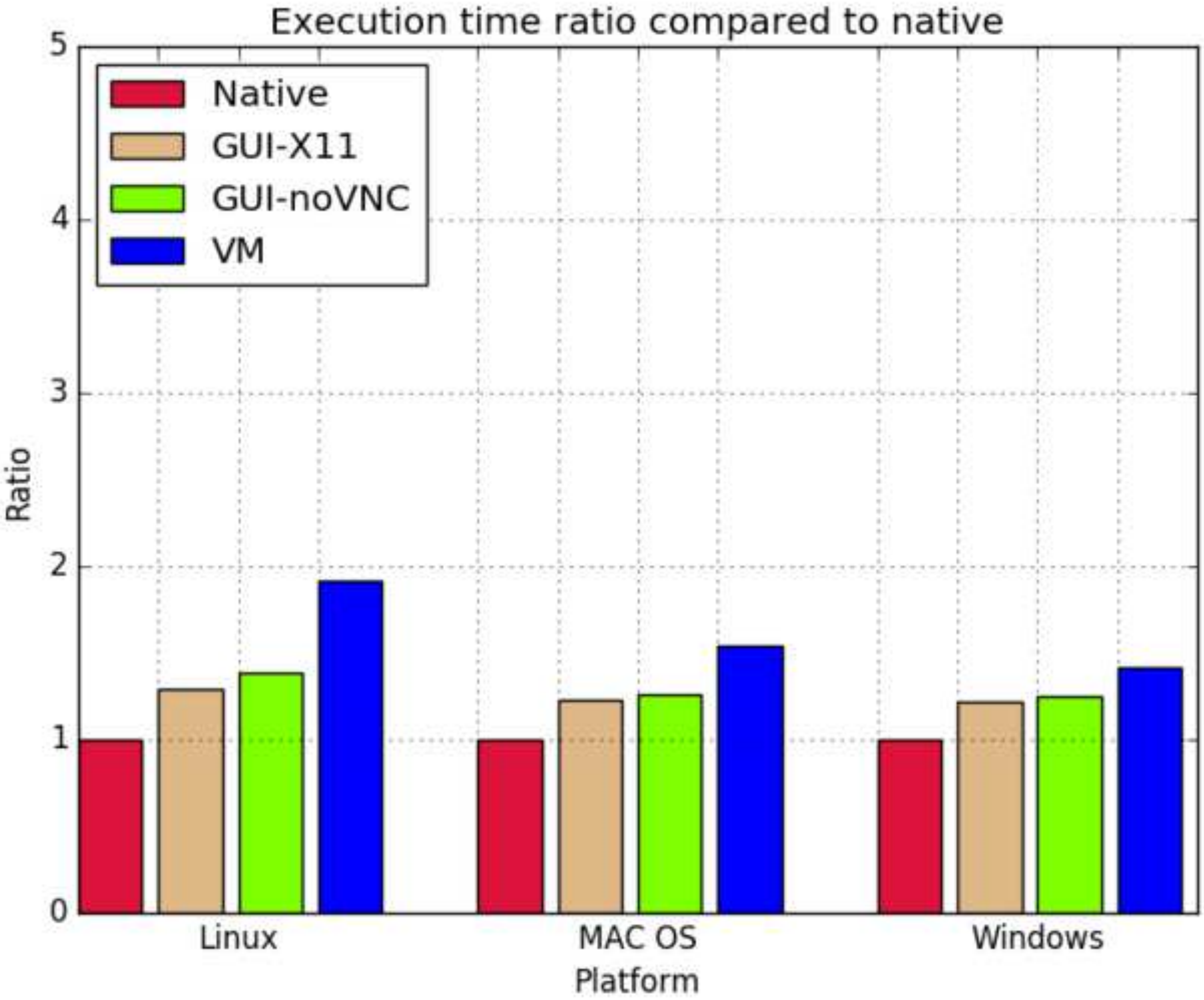

Additional File 1: user manual

Click here to access/download
Supplementary Material
UserManual.pdf

Additional File 2: video of GUIdock-VNC demo

Click here to access/download
Supplementary Material
GUIdock-VNC Demo.mov

Additional File 3: video of deploying GUIdock-VNC on the cloud

Click here to access/download
Supplementary Material
noVNC cloud.mp4

Manuscript (reference pdf)

Click here to access/download
Supplementary Material
bmc_article.pdf
