# Supplementary material for: GUIdock-VNC: using a graphical desktop sharing system to provide a browser-based interface for containerized software
Source: Gigascience. 2017 Feb 24;6(4):1–6. doi: 10.1093/gigascience/giw013 (PMC5530313; doi:10.1093/gigascience/giw013)
Supplement: Supplemental material [file giw013_Supp.zip › UserManual20161111.pdf]

noVNC-CyNetworkBMA

USER MANUAL

Latest version available at https://github.com/BioDepot/GUIdock-VNC

The purpose of this project is to address the issue of delivering graphical interface from Docker container. Our approach is by using the noVNC solution, developed by Kanaka *et. al.* Basically, noVNC is to use browser as VNC client to the running container, giving more freedom and flexibility on having graphical applications running as a docker package.

This Docker container includes: Cytoscape (an open source java application with GUI), R and Bioconductor, and CyNetworkBMA (a Cytoscape plugin to infer gene networks).

This manual demonstrates the installation and instructions to run noVNC.

Docker Image : https://hub.docker.com/r/kristiyanto/novnc-cynetworkbma/
Dockerfile : https://github.com/BioDepot/noVNC-CyNetworkBMA

Authors

Varun Mittal, Ling-Hong Hung, Jayant Keswani, Daniel Kristiyanto, Sung Bong Lee, Ka Yee Yeung

Institute of Technology
University of Washington
Tacoma, WA, 98402, USA

Table of content

1. Docker Installation
    - Linux
    - Mac OS X
    - Windows
2. Running the Cytoscape application
3. On the Cloud
    - Google Cloud
    - AWS
4. Demo

Docker Installation

Linux

Installing Docker

To install docker go to the terminal and follow the steps

1. Update your APT package index.

```
#sudo apt-get update
```

2. Install Docker.

```
#sudo apt-get install docker-engine
```

3. Start the docker daemon.

```
#sudo service docker start
```

4. Verify docker is installed correctly.

```
#sudo docker run hello-world
```

The last command downloads a test image and runs it in a container. When the container runs, it prints an informational message. Then, it exits.

For more information please refer to -
https://docs.docker.com/engine/installation/linux/ubuntulinux/

Mac OS X

Installing Docker

Your Mac must be running OS X 10.8 “Mountain Lion” or newer to run Docker software or If you have Mac OS X 10.10.3 Yosemite or newer, consider using Docker for Mac instead. It runs natively on the Mac, so there is no need for a preconfigured Docker QuickStart shell. It uses xhyve for virtualization, instead of VirtualBox. Full install prerequisites are provided in the Docker for Mac topic in Docker for Mac.

To download to the package - https://www.docker.com/products/docker-toolbox

1. Install Docker Toolbox by double-clicking the package or by right-clicking and choosing “Open” from the pop-up menu.
2. The installer launches an introductory dialog, followed by an overview of what’s installed.
3. Press Continue to install the toolbox.
4. The installer presents you with options to customize the standard installation.
5. By default, the standard Docker Toolbox installation:
    - installs binaries for the Docker tools in `/usr/local/bin`
    - makes these binaries available to all users
    - updates any existing VirtualBox installation
6. For now, don’t change any of the defaults.
7. Press Install to perform the standard installation.
8. The system prompts you for your password.
9. Provide your password to continue with the installation.
10. When it completes, the installer provides you with some shortcuts. You can ignore this for now and click Continue.
11. Then click Close to finish the installer.

To run a Docker container:

- create a new (or start an existing) Docker Engine host running
- switch your environment to your new VM
- use the docker client to create, load, and manage containers

Once you create a machine, you can reuse it as often as you like. Like any Virtual Box VM, it maintains its configuration between uses.

1. Open the Launchpad and locate the Docker Quickstart Terminal icon.
2. Click the icon to launch a Docker Quickstart Terminal window.
3. The terminal does a number of things to set up Docker Quickstart Terminal for you.
4. Click your mouse in the terminal window to make it active.
5. The prompt is traditionally a `$` dollar sign. You type commands into the *command line* which is the area after the prompt. Your cursor is indicated by a highlighted area or a `|` that appears in the command line. After typing a command, always press RETURN.
6. Type the `docker run hello-world` command and press RETURN.

The last command downloads a test image and runs it in a container. When the container runs, it prints an informational message. Then, it exits.

Windows

Installing Docker

If you have a newer system, specifically 64bit Windows 10 Pro, with Enterprise and Education (1511 November update, Build 10586 or later), consider using Docker for Windows instead. It runs natively on the Windows, so there is no need for a preconfigured Docker QuickStart shell. It also uses Hyper-V for virtualization.

To download to the package - https://www.docker.com/products/docker-toolbox

1. Go to folder where the installation file is saved and run the installation file.
2. Click the installer link to download.
3. Install Docker Toolbox by double-clicking the installer.
4. The installer launches the "Setup - Docker Toolbox" dialog.
5. If Windows security dialog prompts you to allow the program to make a change, choose Yes. The system displays the Setup - Docker Toolbox for Windows Wizard.
6. Press Next to accept all the defaults and then Install.
7. Accept all the installer defaults. The installer takes a few minutes to install all the components:
8. When notified by Windows Security the installer will make changes, make sure you allow the installer to make the necessary changes.
9. When it completes, the installer reports it was successful:

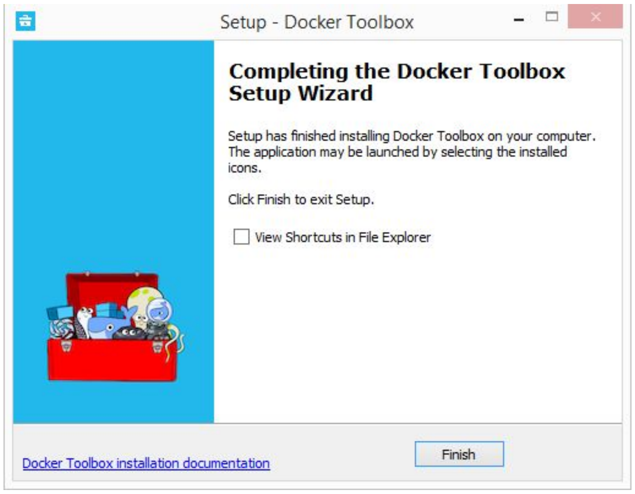

10. Uncheck “View Shortcuts in File Explorer” and press Finish.

To run a Docker container:

The installer places Docker Toolbox and VirtualBox in your Applications folder. In this step, you start Docker Toolbox and run a simple Docker command.

1. On your Desktop, find the Docker Toolbox icon.

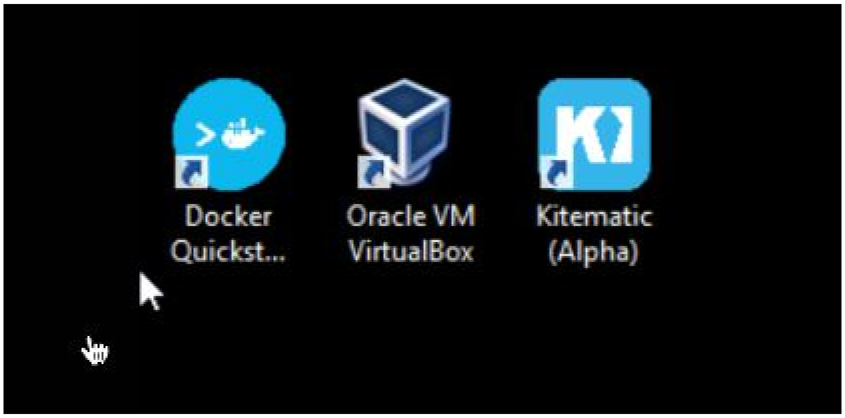

2. Click the icon to launch a Docker Toolbox terminal.
3. If the system displays a User Account Control prompt to allow VirtualBox to make changes to your computer. Choose Yes.
4. The terminal does several things to set up Docker Toolbox for you. When it is done, the terminal displays the `$` prompt.

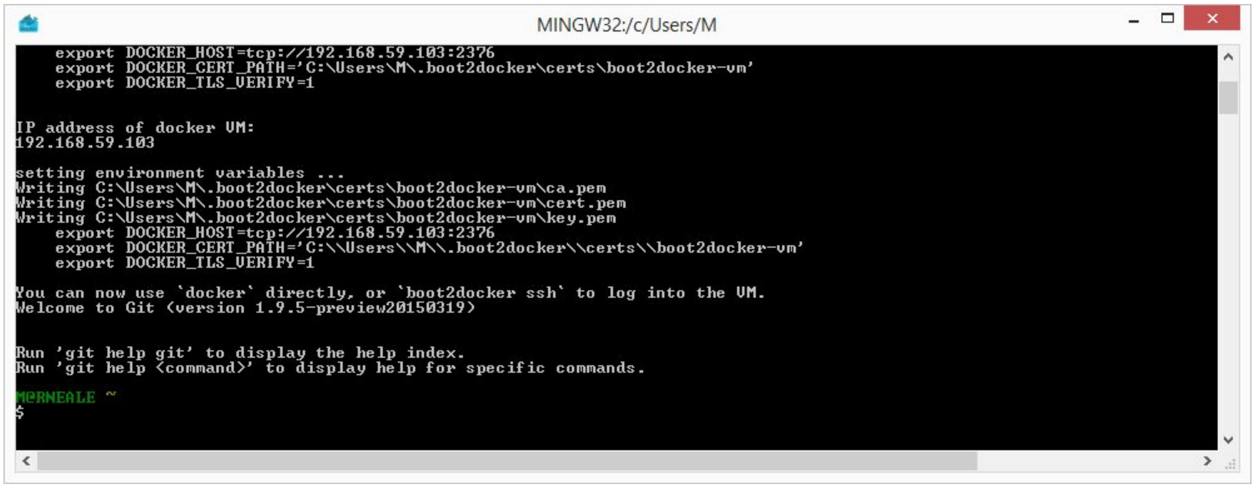

5. The terminal runs a special `bash` environment instead of the standard Windows command prompt. The `bash` environment is required by Docker.
6. Make the terminal active by click your mouse next to the `$` prompt.

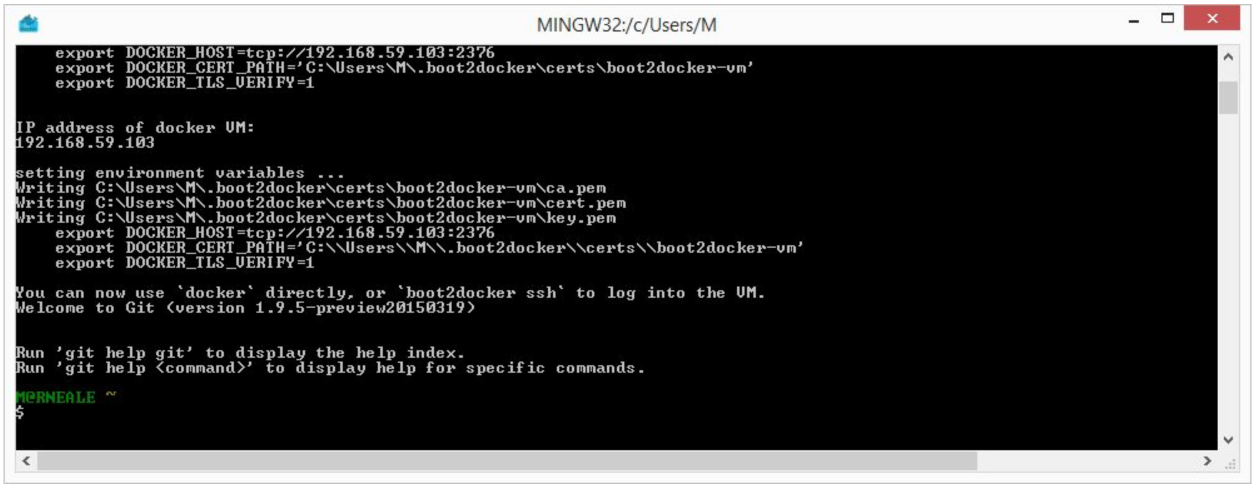

7. The prompt is traditionally a `$` dollar sign. You type commands into the *command line* which is the area after the prompt. Your cursor is indicated by a highlighted area or a `|` that appears in the command line. After typing a command, always press RETURN.
8. Type the `docker run hello-world` command and press RETURN.

The last command downloads a test image and runs it in a container. When the container runs, it prints an informational message. Then, it exits.

Running the Cytoscape application

After you have installed docker on your machine, launch docker and type the following commands on Docker terminal to install and run the Cytoscape application (same across all the platforms)

1. Downloading the image
   `# docker pull biodepot/novnc-cynetworkbma`

```
MacBook-Air:~ jayant$ docker pull biodepot/novnc-cynetworkbma
Using default tag: latest
latest: Pulling from biodepot/novnc-cynetworkbma

56eb14001ceb: Already exists
7ff49c327d83: Already exists
6e532f87f96d: Already exists
3ce63537e70c: Already exists
e319c61017b1: Downloading [===========>                                   ] 4.796 MB/21.75 MB
de2f07c0daa2: Downloading [==================>                            ] 4.583 MB/12.42 MB
62f78686c1db: Download complete
15416a3098e0: Download complete
1a5b1f623cbd: Waiting
addbdca52169: Waiting
7d6cf4475f54: Waiting
4adbe653f4d9: Waiting
09d6505b3a3a: Waiting
053b440e7dc4: Waiting
9e754ce2784f: Waiting
402ecf9623e7: Waiting
182ad1d36955: Waiting
58877bd66241: Waiting
```

2. Running the application
   `# docker run -v /Users/path/to/your/files:/data -d -p 80:6080 biodepot/novnc-cynetworkbma`

Once the container is running you can open any of the browser to interact with it by using the default noVNC port 32768 or you can check the port and IP at which the service is running by using docker command – `# docker ps`

```
MacBook-Air:~ jayant$ docker ps
CONTAINER ID        IMAGE                                   COMMAND
CREATED             STATUS              PORTS                     NAMES
706e1aed70c3        biodepot/novnc-cynetworkbma:benchmark   "/entrypoint.sh"
4 seconds ago       Up 3 seconds        0.0.0.0:32768->6080/tcp   small_jang
8fd09521d4b1        biodepot/novnc-cynetworkbma             "/entrypoint.sh"
2 minutes ago       Up 2 minutes        0.0.0.0:80->6080/tcp      sleepy_austin
MacBook-Air:~ jayant$
```

Type the URL http://dockerMachineIPAddress:noVNCport on the browser and start the application by going Menu -> Other -> Cytoscape

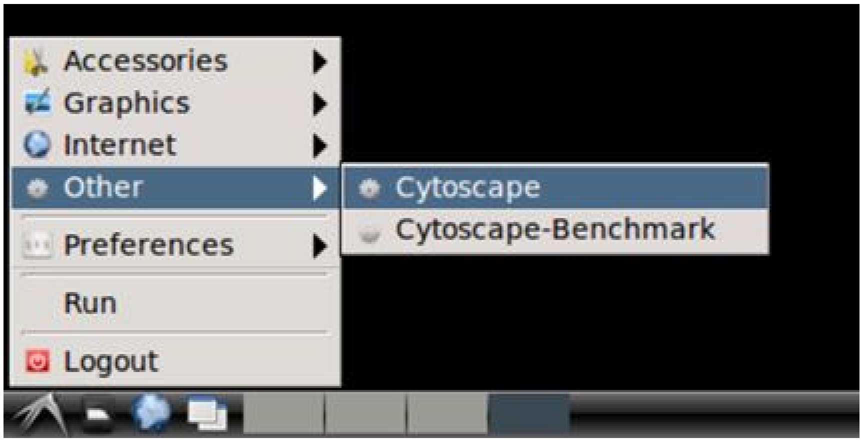
Accessories
Graphics
Internet
Other
Cytoscape
Cytoscape-Benchmark
Preferences
Run
Logout

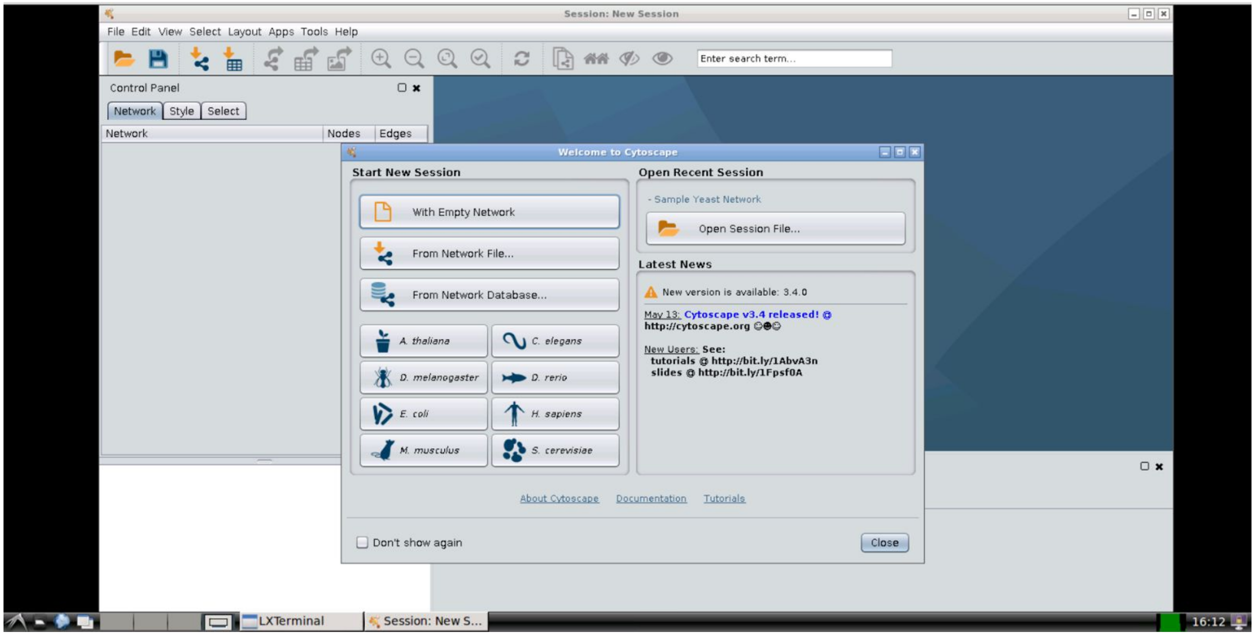
Session: New Session
File Edit View Select Layout Apps Tools Help
Enter search term...
Control Panel
Network Style Select
Network Nodes Edges
Welcome to Cytoscape
Start New Session
With Empty Network
From Network File...
From Network Database...
A. thaliana
C. elegans
D. melanogaster
D. rerio
E. coli
H. sapiens
M. musculus
S. cerevisiae
Open Recent Session
- Sample Yeast Network
Open Session File...
Latest News
New version is available: 3.4.0
May 13: Cytoscape v3.4 released!
http://cytoscape.org
New Users: See:
tutorials @ http://bit.ly/1AbvA3n
slides @ http://bit.ly/1Fpsf0A
About Cytoscape
Documentation
Tutorials
Don't show again
Close
LXTerminal
Session: New S...
16:12

On The Cloud

Google Cloud using Api

1. Login to your google cloud console
2. Select credentials from the pane of on the left
3. Create a new credential (Oauth client ID)
4. Create consent screen
5. Give it a name and set Authorized javascript origin to http://vm1.com and set Authorized redirect URL's to http://vm1.com/auth/google_authenticate.
6. After saving the configuration your client ID and client secret key will appear, save them.
7. Open the terminal on your machine and run the command
```
#Docker run -td -p 0.0.0.0:6080 --name demo1 biodepot/novnc-cynetworkbma:benchmark -u google.client.id -p google.client.secret -e gmail.address
```
8. Open the browser on your machine and type http://vm1.com
9. You will be redirected to google authentication, authenticate it with your email ID and password and you will be have the access to GUIdock-vnc container
10. Select the cytoscape application from the start menu

Amazon AWS

1. Login to your console and create a new EC2 instance of ubuntu (Here we are using ubuntu you can choose operating system of your choice)
2. Select the configuration and click on "Review and Launch"
3. You will be prompted to associate a ssh key pair with the instance, you can use an existing key pair or create a new one. The key will be downloaded onto the computer which will be later used to ssh into the machine.
4. Once the instance is running select your instance and scroll right for security groups.

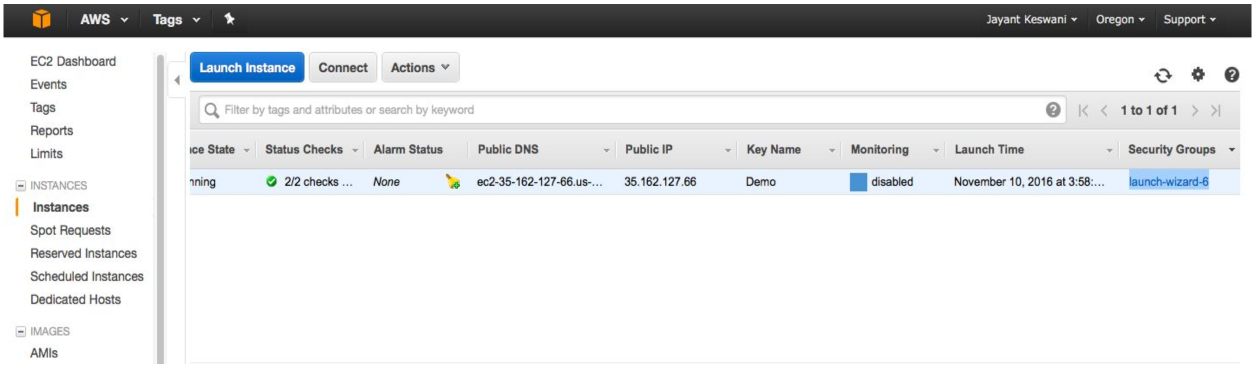

5. From "Actions" button select "Edit inbound rules"

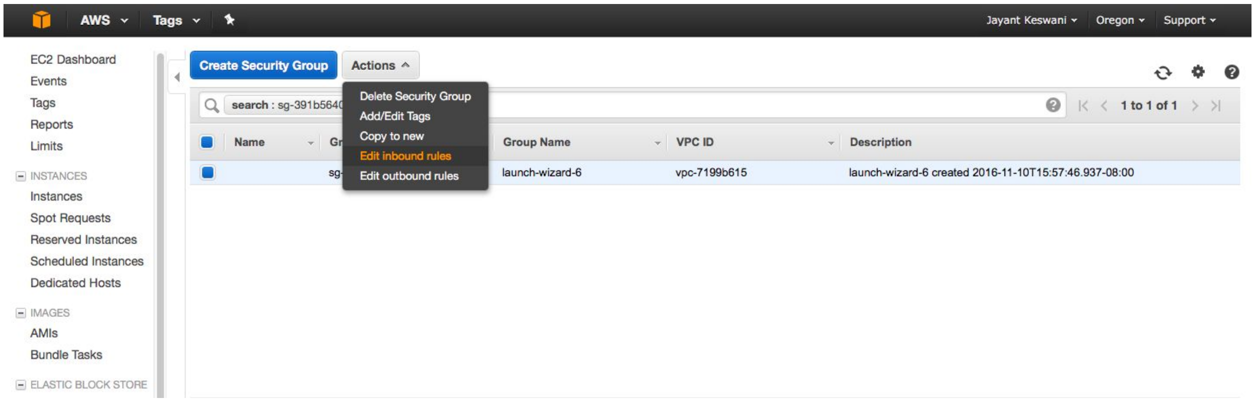

6. Add a new http rule for port 6080 to access the GUI from the container

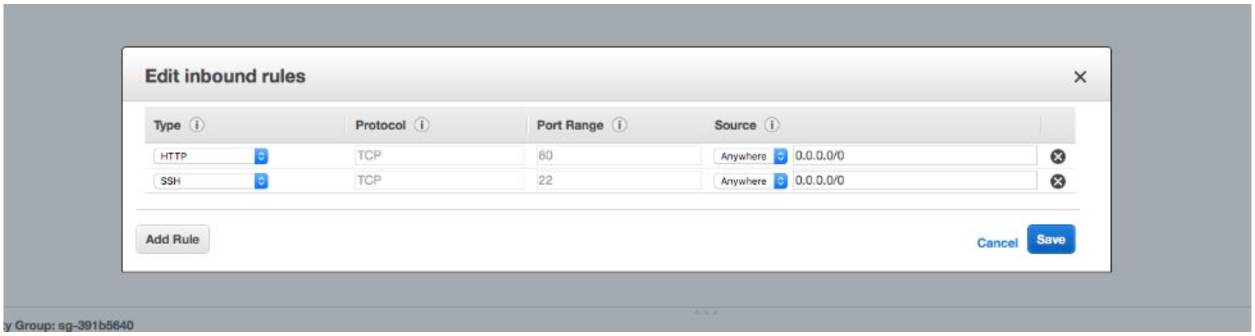

7. Copy the public dns of the instance

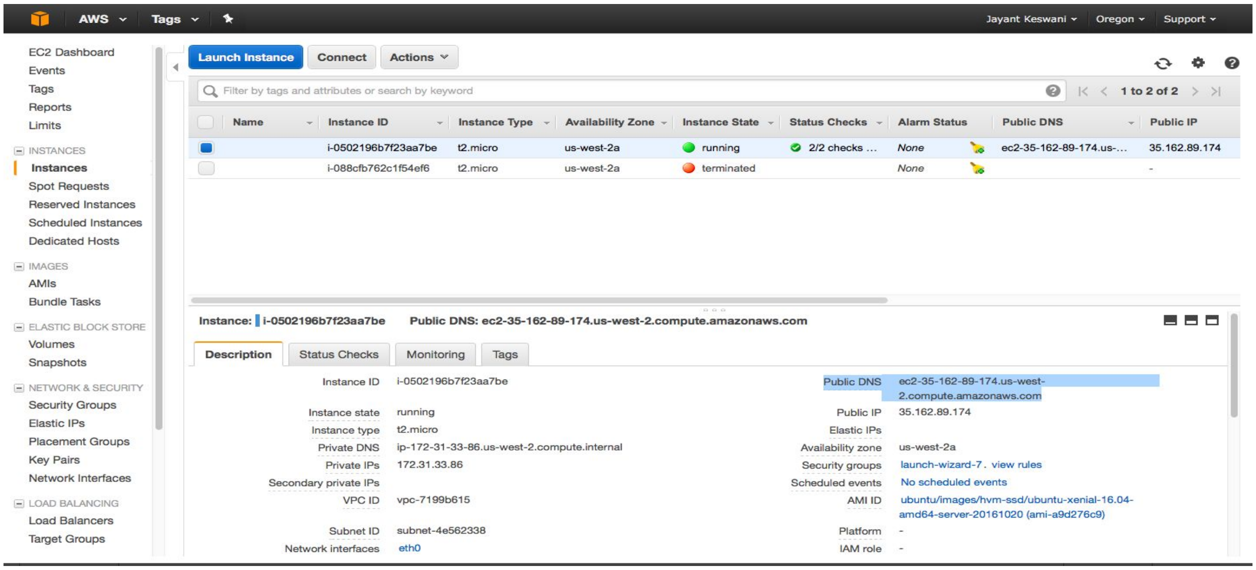

8. SSH into the instance by typing the following command into the terminal.
(Type the commands in the directory where the ssh key of AWS instance was downloaded)

```
# chmod 400 demo.pen (demo.pen is name of the key)
# ssh -i demo.pen ubuntu@public-dns-of-aws-instance
```

9. After you are logged in type these commands to install docker on the instance

```
# sudo apt-get update
# sudo apt-get install docker.io
#Docker run -td -p 0.0.0.0:6080 --name demo1 biodepot/novnc-cynetworkbma:benchmark -u google.client.id -p google.client.secret -e gmail.address
```

10. Use your favorite browser and go to the address to access the container

```
public-dns-of-aws-instance:6080
public-ip-of aws-instance:6080
```

11. Select the cytoscape application from the start menu

On the cloud, GUIdock-NoVNC can also be run on any cloud instances. Please refer to the Linux and Windows to install GUIdock-NoVNC on the cloud corresponds with the operating system installed for the cloud instance.

Firewall Configurations

Please refer to the following documentations for more information:

- Google Cloud Platform: https://cloud.google.com/compute/docs/networking
- Amazon Web Services: http://docs.aws.amazon.com/AWSEC2/latest/UserGuide/authorizing-access-to-an-instance.html
- Microsoft Azure: https://azure.microsoft.com/en-us/documentation/articles/virtual-machines-windows-classic-setup-endpoints/

On Distributed Docker Platform

GUIdock-NoVNC can also be run on a distributed Docker Platform (e.g. Google Container, Docker Swarm, or Amazon EC2 Container Service). GUIdock-NoVNC is not designed for distributed system, hence, running multiple container does not increase the container's performance --instead each single containers serve as an independent container. Accessing the container can be performed using the container's public IP address from a web browser.

Demo

Here is the link to the -- demo
