# Supplementary figures and images for: GUIdock-VNC: using a graphical desktop sharing system to provide a browser-based interface for containerized software
Source: Gigascience. 2017 Feb 24;6(4):1–6. doi: 10.1093/gigascience/giw013 (PMC5530313; doi:10.1093/gigascience/giw013)

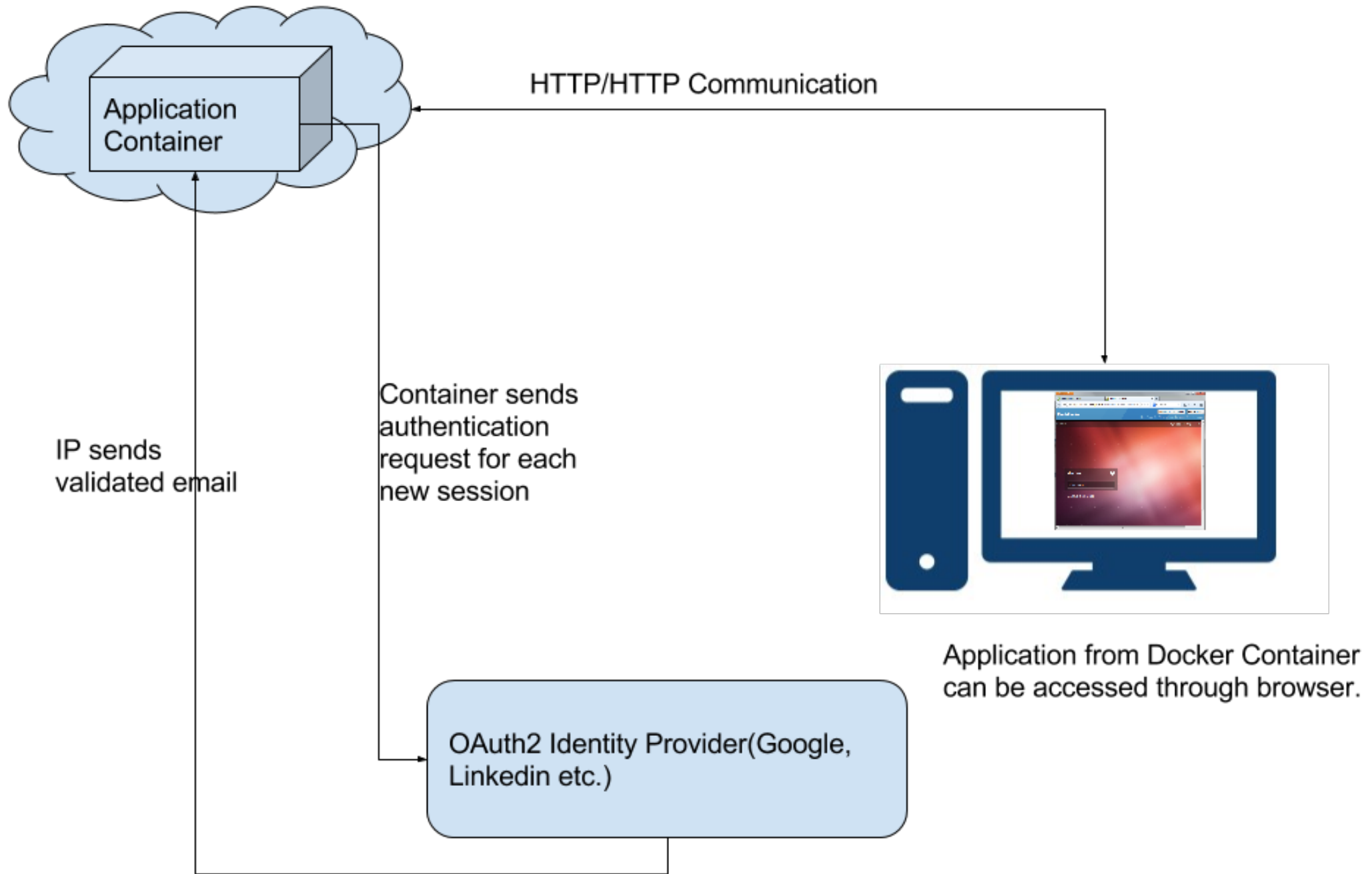

Supplement: Supplemental material [file giw013_Supp.zip › Architecture.pdf]

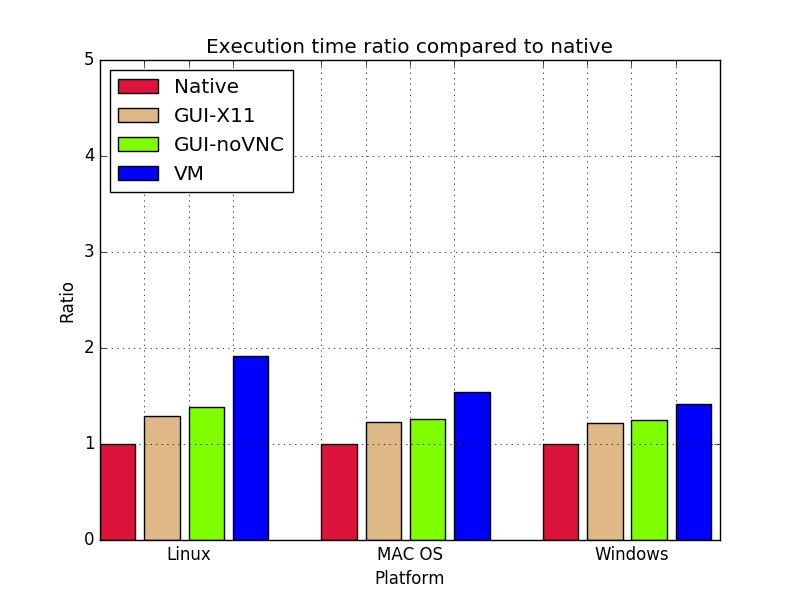

Supplement: Supplemental material [file giw013_Supp.zip › execRatios2.png]

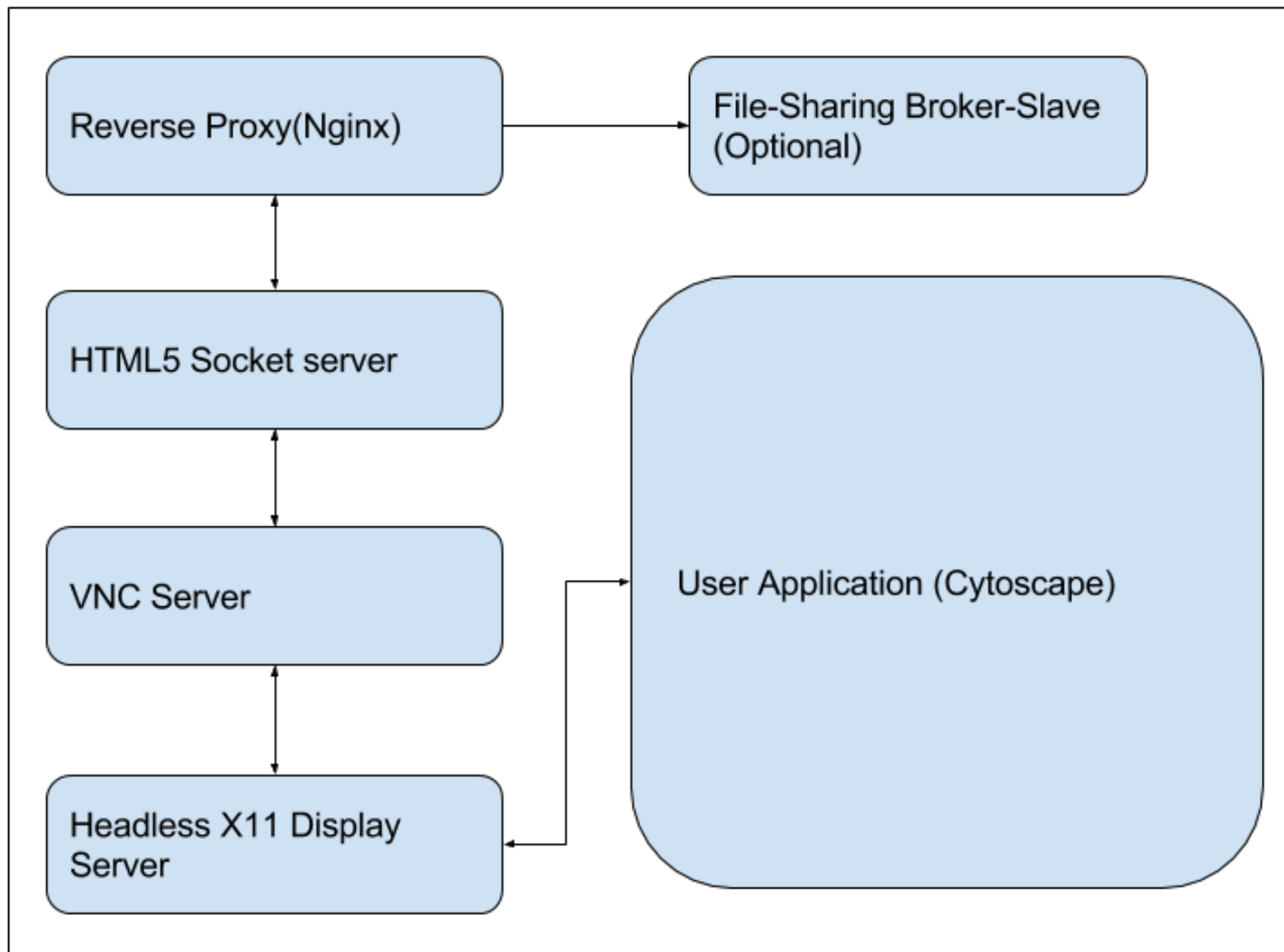

Docker Application Image

Supplement: Supplemental material [file giw013_Supp.zip › Infrastructure.pdf]

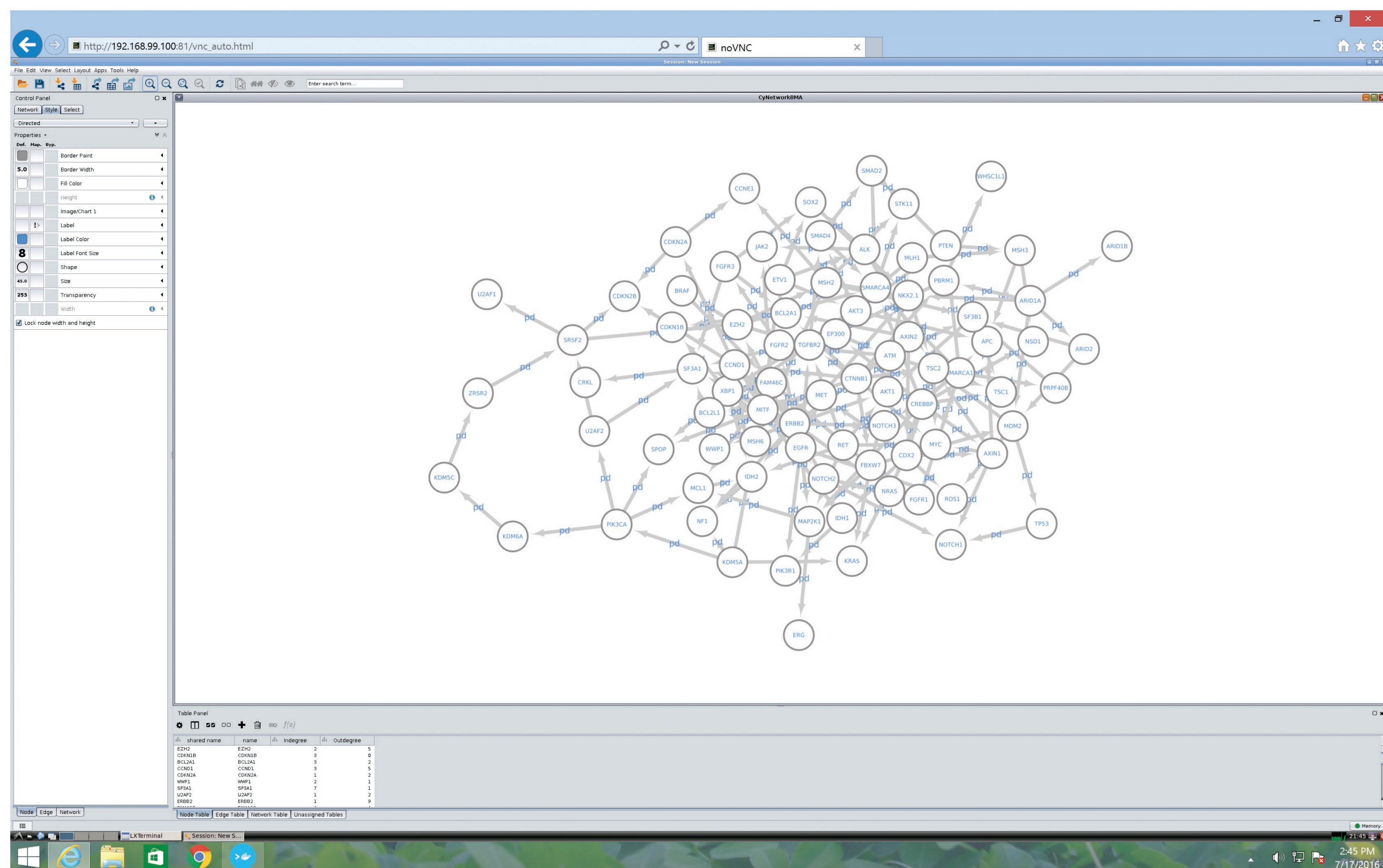

(a)

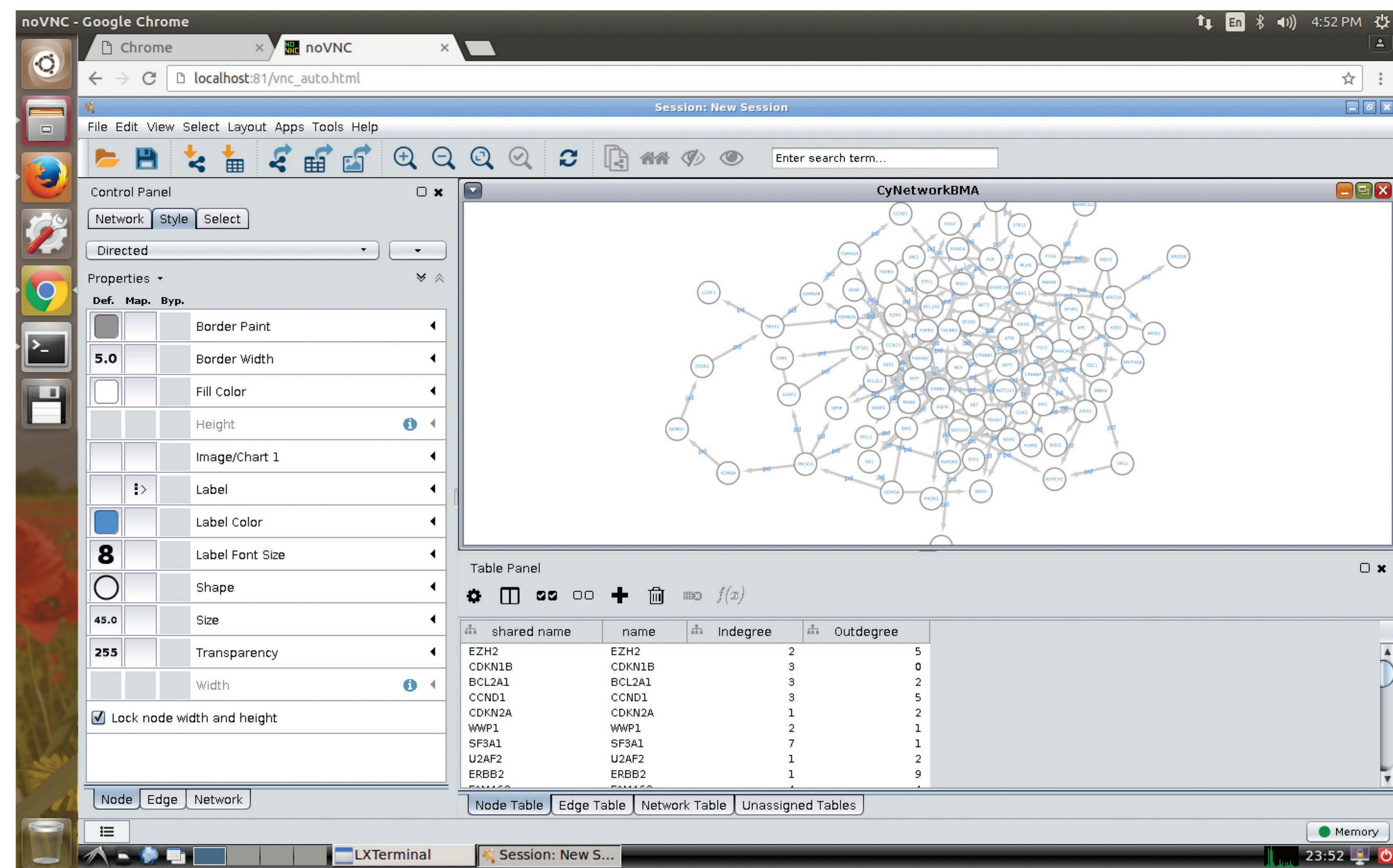

(b)

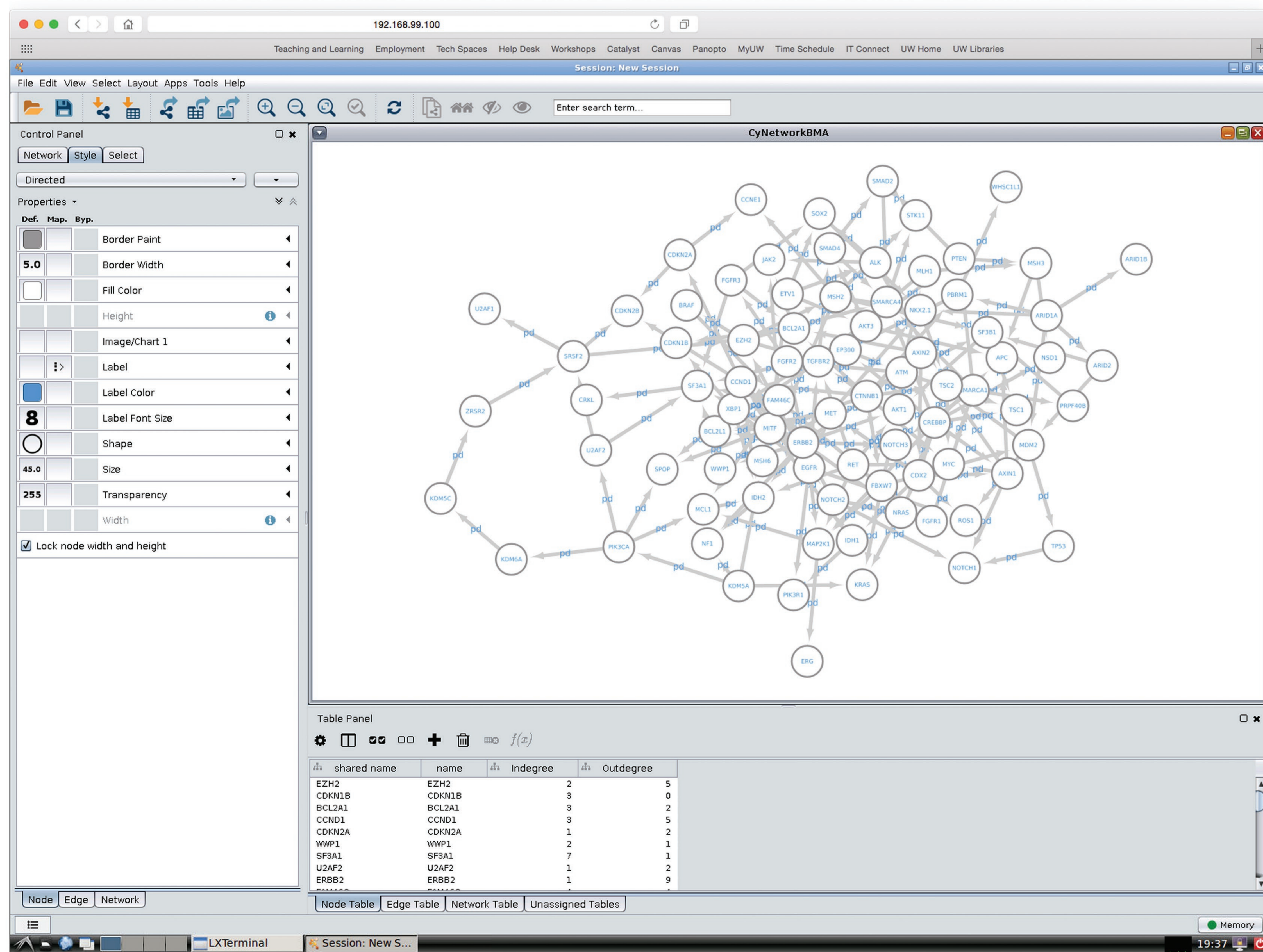

(c)

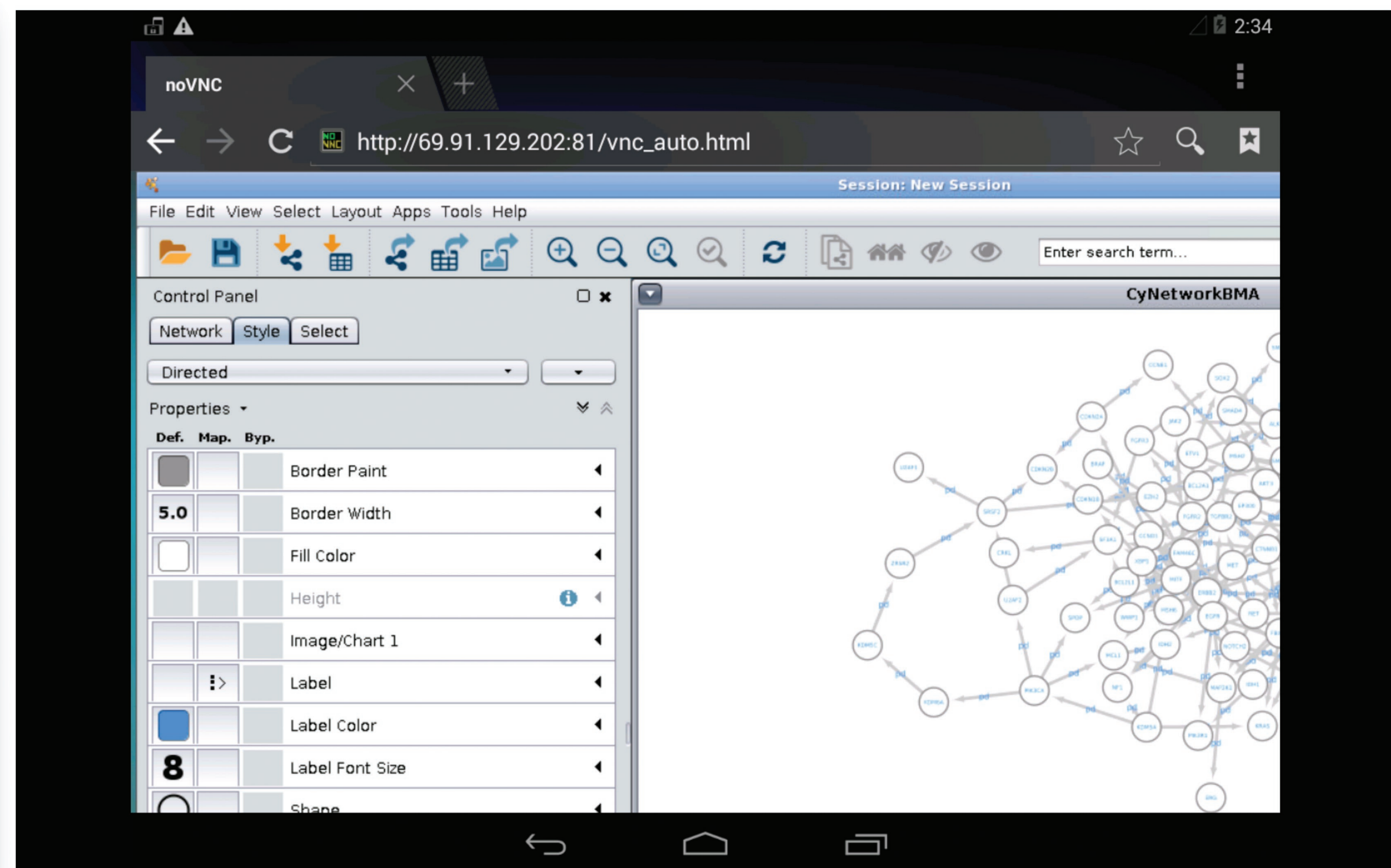

(d)

Supplement: Supplemental material [file giw013_Supp.zip › Screenshots.pdf]
