# Supplementary material for: GUIdock-VNC: using a graphical desktop sharing system to provide a browser-based interface for containerized software
Source: Gigascience. 2017 Feb 24;6(4):1–6. doi: 10.1093/gigascience/giw013 (PMC5530313; doi:10.1093/gigascience/giw013)
Supplement: Response_to_Reviewer_Comments_Original_Submission.pdf [file giw013_Response_to_Reviewer_Comments_Original_Submission.pdf]

We are very grateful for the comments and feedback from the reviewers. We are particularly appreciative that the reviewers tested our tool GUIdock-VNC.

We addressed all of the comments in this revision.

Reviewers' comments:
REVIEWER #1: Page 11 line 33 - "passon" should be "pass on"

RESPONSE: We fixed this typo on line 217 (page 11).

REVIEWER #2: Given the aim of making this tool available - was there an issue with MS Internet Explorer (I note that Edge's HTML5 support is mentioned but is then not listed in browsers supported under Availability and requirements)

RESPONSE: After we submitted this manuscript, we saw that Microsoft has expanded the support of html5 on Edge. We observed that Edge works for Windows 10 Pro using Docker for Windows, and with pop-up blocker off. However, Edge does not work for Windows 10 Home. We added this to lines 244-245 on page 12.

REVIEWER #2: It would be nice to have had videos of the use case being deployed to the other cloud environments (Azure & AWS for example were mentioned as having been tested).

RESPONSE: We expanded the user manual (Additional File 1) to include instructions of deploying on the cloud (see pages 10, 11, 12 in Additional File 1).
