# Supplementary material for: GUIdock-VNC: using a graphical desktop sharing system to provide a browser-based interface for containerized software
Source: Gigascience. 2017 Feb 24;6(4):1–6. doi: 10.1093/gigascience/giw013 (PMC5530313; doi:10.1093/gigascience/giw013)
Supplement: Response_to_Reviewer_Comments_Revision_1.pdf [file giw013_Response_to_Reviewer_Comments_Revision_1.pdf]

We added the "Availability of supporting data" section and reference [22] as required on the letter dated Dec 13.

In addition, we checked the mockup of the DOI. Everything looks great. Please go ahead to switch it live.

Thank you for your consideration.
Ka Yee Yeung
Associate Professor, University of Washington Tacoma
