# Supplementary material for: GUIdock-VNC: using a graphical desktop sharing system to provide a browser-based interface for containerized software
Source: Gigascience. 2017 Feb 24;6(4):1–6. doi: 10.1093/gigascience/giw013 (PMC5530313; doi:10.1093/gigascience/giw013)
Supplement: Reviewer_1_Report_(Original_Submission).pdf [file giw013_Reviewer_1_Report_(Original_Submission).pdf]

Reviewer Report

Title: "GUIdock-VNC: Using a graphical desktop sharing system to provide a browser-based interface for containerized software"

Version: Original Submission **Date:** 10/3/2016

Reviewer name: Peter Li

Reviewer Comments to Author:

This well-written manuscript is a report on GUIdock-VNC, a layer of software for use inside Docker containers to allow bioinformatics applications to export a GUI via a web browser using the VNC protocol. A use case is also presented showing how GUIdock-VNC can be applied to a bioinformatics tool called CyNetworkBMA, allowing users to interact with its Cytoscape GUI from within a web browser to load interaction data and infer gene networks from it.

From a reproducibility point of view, I feel that the authors have done an excellent job in providing the documentation for me to use the CyNetworkBMA application through GUIdock-VNC. I was able to run CyNetworkBMA locally on my Mac Pro desktop PC using Docker Tool with their biodepot/novnc-cynetworkbma Docker image. In addition, I was also able to deploy their Docker image on the AWS cloud using an EC2 virtual server running Ubuntu and access the CyNetworkBMA Cytoscape GUI on a web browser from my office computer.

The authors cite their previous work published in PLOS ONE earlier this year on GUIdock which, like the current manuscript, is also a framework for delivering GUI applications from Docker containers and its use has been demonstrated with the CyNetworkBMA application. The difference is that GUIdock uses an X11 implementation so I did wonder if the new GUIdock VNC implementation is sufficiently new work which merits publication in GigaScience? The authors also compare and contrast the differences between GUIdock-X11 and GUIdock-VNC in the current manuscript, looking at issues such as security, network performance and cloud deployment. Since I think that GUIdock-VNC is a well-presented piece of work, I am inclined to accept it for publication but I suggest that the editors consider the same issue I had.

Manuscript correction

Page 11 line 33 - "passon" should be "pass on"

Level of Interest

Please indicate how interesting you found the manuscript: An article whose findings are important to those with closely related research interests

Quality of Written English

Please indicate the quality of language in the manuscript: Acceptable

Declaration of Competing Interests

Please complete a declaration of competing interests, considering the following questions:

- Have you in the past five years received reimbursements, fees, funding, or salary from an organisation that may in any way gain or lose financially from the publication of this manuscript, either now or in the future?
- Do you hold any stocks or shares in an organisation that may in any way gain or lose financially from the publication of this manuscript, either now or in the future?
- Do you hold or are you currently applying for any patents relating to the content of the manuscript?
- Have you received reimbursements, fees, funding, or salary from an organization that holds or has applied for patents relating to the content of the manuscript?
- Do you have any other financial competing interests?
- Do you have any non-financial competing interests in relation to this paper?

If you can answer no to all of the above, write 'I declare that I have no competing interests' below. If your reply is yes to any, please give details below.

I declare that I have no competing interests.

I agree to the open peer review policy of the journal. I understand that my name will be included on my report to the authors and, if the manuscript is accepted for publication, my named report including any attachments I upload will be posted on the website along with the authors' responses. I agree for my report to be made available under an Open Access Creative Commons CC-BY license (http://creativecommons.org/licenses/by/4.0/). I understand that any comments which I do not wish to be included in my named report can be included as confidential comments to the editors, which will not be published.

I agree to the open peer review policy of the journal
