# Supplementary material for: GUIdock-VNC: using a graphical desktop sharing system to provide a browser-based interface for containerized software
Source: Gigascience. 2017 Feb 24;6(4):1–6. doi: 10.1093/gigascience/giw013 (PMC5530313; doi:10.1093/gigascience/giw013)
Supplement: Reviewer_2_Report_(Original_Submission).pdf [file giw013_Reviewer_2_Report_(Original_Submission).pdf]

Reviewer Report

Title: "GUIdock-VNC: Using a graphical desktop sharing system to provide a browser-based interface for containerized software"

Version: Original Submission **Date:** 10/6/2016

Reviewer name: Mark Fernandes

Reviewer Comments to Author:

Very interesting paper that identifies a niche in Docker container-based applications and builds upon earlier efforts.

The manuscript successfully addresses issues such as effects upon execution speed, Ability to deploy to Cloud with

authentication and software environment requirements (i.e. upon Windows & MacOS).

I particularly approved of the choice of application for the use case. Cytoscape can be a very resource-hungry

application and clearly could benefit from deployment to the Cloud for execution as the client does not then

require those hardware resources (especially for occasional use). This 'lowers the bar' to researchers seeking

to reproduce published research.

Given the aim of making this tool available - was there an issue with MS Internet Explorer (I note that Edge's

HTML5 support is mentioned but is then not listed in browsers supported under Availability and requirements)

The supplemental video demonstrating Cloud deployment is welcomed (I note a minor typo which should have been

Demonstration in the legend). It would be nice to have had videos of the use case being deployed to the other

cloud environments (Azure & AWS for example were mentioned as having been tested).

I found your paper very readable and I think that it will be understandable by readers from a wide range of

levels of experience in this field.

I have visited the projects GitHub and look forward to evaluating this project for use in bespoke Bioinformatics training environments.

Level of Interest

Please indicate how interesting you found the manuscript: An article of importance in its field

Quality of Written English

Please indicate the quality of language in the manuscript: Acceptable

Declaration of Competing Interests

Please complete a declaration of competing interests, considering the following questions:

- Have you in the past five years received reimbursements, fees, funding, or salary from an organisation that may in any way gain or lose financially from the publication of this manuscript, either now or in the future?
- Do you hold any stocks or shares in an organisation that may in any way gain or lose financially from the publication of this manuscript, either now or in the future?
- Do you hold or are you currently applying for any patents relating to the content of the manuscript?
- Have you received reimbursements, fees, funding, or salary from an organization that holds or has applied for patents relating to the content of the manuscript?
- Do you have any other financial competing interests?
- Do you have any non-financial competing interests in relation to this paper?

If you can answer no to all of the above, write 'I declare that I have no competing interests' below. If your reply is yes to any, please give details below.

I declare that I have no competing interests

I agree to the open peer review policy of the journal. I understand that my name will be included on my report to the authors and, if the manuscript is accepted for publication, my named report including any attachments I upload will be posted on the website along with the authors' responses. I agree for my report to be made available under an Open Access Creative Commons CC-BY license (http://creativecommons.org/licenses/by/4.0/). I understand that any comments which I do not wish to be included in my named report can be included as confidential comments to the editors, which will not be published.

I agree to the open peer review policy of the journal
